# Kihi-to, a herbal traditional medicine, improves Abeta(25–35)-induced memory impairment and losses of neurites and synapses

**DOI:** 10.1186/1472-6882-8-49

**Published:** 2008-08-16

**Authors:** Chihiro Tohda, Rie Naito, Eri Joyashiki

**Affiliations:** 1 Division of Biofunctional Evaluation, Research Center for Ethnomedicine, Institute of Natural Medicine, University of Toyama, Toyama 930-0194, Japan

## Abstract

**Background:**

We previously hypothesized that achievement of recovery of brain function after the injury requires the reconstruction of neuronal networks, including neurite regeneration and synapse reformation. Kihi-to is composed of twelve crude drugs, some of which have already been shown to possess neurite extension properties in our previous studies. The effect of Kihi-to on memory deficit has not been examined. Thus, the goal of the present study is to determine the *in vivo *and *in vitro *effects of Kihi-to on memory, neurite growth and synapse reconstruction.

**Methods:**

Effects of Kihi-to, a traditional Japanese-Chinese traditional medicine, on memory deficits and losses of neurites and synapses were examined using Alzheimer's disease model mice. Improvements of Aβ(25–35)-induced neuritic atrophy by Kihi-to and the mechanism were investigated in cultured cortical neurons.

**Results:**

Administration of Kihi-to for consecutive 3 days resulted in marked improvements of Aβ(25–35)-induced impairments in memory acquisition, memory retention, and object recognition memory in mice. Immunohistochemical comparisons suggested that Kihi-to attenuated neuritic, synaptic and myelin losses in the cerebral cortex, hippocampus and striatum. Kihi-to also attenuated the calpain increase in the cerebral cortex and hippocampus. When Kihi-to was added to cells 4 days after Aβ(25–35) treatment, axonal and dendritic outgrowths in cultured cortical neurons were restored as demonstrated by extended lengths of phosphorylated neurofilament-H (P-NF-H) and microtubule-associated protein (MAP)2-positive neurites. Aβ(25–35)-induced cell death in cortical culture was also markedly inhibited by Kihi-to. Since NF-H, MAP2 and myelin basic protein (MBP) are substrates of calpain, and calpain is known to be involved in Aβ-induced axonal atrophy, expression levels of calpain and calpastatin were measured. Treatment with Kihi-to inhibited the Aβ(25–35)-evoked increase in the calpain level and decrease in the calpastatin level. In addition, Kihi-to inhibited Aβ(25–35)-induced calcium entry.

**Conclusion:**

In conclusion Kihi-to clearly improved the memory impairment and losses of neurites and synapses.

## Background

Neuronal death, neuritic atrophy, and loss of synapses underlie the pathogenesis of Alzheimer's [1-3]. However, neurons with atrophic neurites may remain viable and have the potential to remodel, even when neuronal death has occurred in other parts of the brain. We previously hypothesized that achievement of recovery of brain function after the injury requires the reconstruction of neuronal networks, including neurite regeneration and synapse reformation [4]. Among several traditional Chinese medicines, Ginseng Radix [5,6], Astragali Radix [7], and Polygalae Radix [8] showed axonal extension activity after amyloid β (Aβ) (25–35)-induced axonal atrophy. Further, ginsenoside Rb_1_, a constituent of Ginseng Radix, and the aqueous extract of Astragali Radix attenuated spatial memory deficits and the loss of axons and synapses in the brain of Aβ(25–35)-injected mice. Although cholinesterase inhibitors, such as donepezil hydrochloride, are clinically used for Alzheimer's disease, they do not prevent or reverse the underlying neurodegeneration [9] or attenuate impairments in memory and neuronal damage in Aβ(25–35)-injected mice [6,10].

Aβ(25–35) can be produced in Alzheimer's disease patients by enzymatic cleavage of the naturally occurring Aβ(1–40) [11]. Abundant reports support that Aβ(25–35) is an active partial fragment of amyloid β. This fragment also forms a β-sheet structure [12] and induces neuronal cell death [12,13], neuritic atrophy [9], synaptic loss [6,10,14]. Moreover, our previous work also demonstrated that Aβ(25–35) and Aβ(1–42) resulted in similar effects on neuritic atrophy and cell death at 10 μM [15]. Furthermore, a recent report showed that a single intracerebroventricular (i.c.v., 15 μg) injection of Aβ(25–35) could induce major neuropathological signs related to early stages of Alzheimer's disease in rats [16].

Kihi-to is a herbal drug used in the Japanese-Chinese traditional medicine. Kihi-to was described to be effective for insomnia, anemia, amnesia, depression, and neurosis in classical literatures. However, basic researches of Kihi-to against dementia have been very few yet. Only one meeting report described that 25 patients with senile dementia improved Mini-Mental State Examination score after 3 months administration of Kihi-to [17].

Kihi-to is composed of twelve crude drugs, some of which (e.g., Ginseng Radix [5,6], Astragali Radix [7] and Polygalae Radix [8]) have already been shown to possess neurite extension properties in our previous studies. Although a previous study by another group has demonstrated that choline acetyltransferase activity is up-regulated by Kihi-to in rat embryo septal cultures [18], the effect of Kihi-to on memory deficit has not been examined. Thus, the goal of the present study is to determine the *in vivo *and *in vitro *effects of Kihi-to on memory, neurite growth and synapse reconstruction.

## Methods

### Materials

A partial fragment of Aβ, Aβ(25–35) (Sigma-Aldrich, Saint Louis, MO, USA), was dissolved in sterile distilled water (in vitro experiments) or physiological saline (in vivo experiments) at a concentration of 5 mM and was incubated at 37°C for 4 days to allow fibril formation. A reverse fragment, Aβ(35–35) (Sigma-Aldrich) was also dissolved in physiological saline (in vivo experiments) at a concentration of 5 mM and was incubated at 37°C for 4 days to allow fibril formation. Neurobasal media and B-27 supplement were purchased from Gibco BRL (Rockville, MD, USA). Mouse β-NGF was purchased from Astral Biologicals (San Ramon, CA, USA). A monoclonal antibody against phosphorylated neurofilament-H (NF-H) was purchased from Sternberger Monoclonals Incorporated (Lutherville, MD, USA). Monoclonal and polyclonal antibodies against microtubule-associated protein 2a and 2b (MAP2), a monoclonal antibody against synaptophysin, a polyclonal antibody against myelin basic protein (MBP) were purchased from Chemicon (Temecula, CA, USA). A monoclonal antibody against μ-calpain and a polyclonal antibody against calpastatin were purchased from Biosource (Camarillo, CA, USA) and Santa Cruz Biotechnology (Santa Cruz CA. USA), respectively. MDL28170 was purchased from Biomol (Plymouth Meeting, PA, USA). Alexa Fluor 488-conjugated goat anti-mouse IgG and Alexa Fluor 568-conjugated goat anti-rabbit IgG were purchased from Molecular Probes (Eugene, OR, USA).

### Preparation of Kihi-to extract

Kihi-to is composed of twelve types of crude drugs: Ginseng Radix (*P. ginseng *C.A. Meyer), 3 g; Polygalae Radix (*P. tenuifolia *Willd.), 2 g; Astragali Radix (*A. membranaceus *Bunge), 3 g; Zizyphi Fructus (*Zizyphus jujube *Mill. var. *inermis *Rehd.) 2 g; Zizyphi Spinosi Semen (*Z. jujube *Mill. var. spinosa (Bunge) Hu ex H.F. Chou) 3 g; Angelicae Radix (*Angelica acutiloba *Kitagawa) 2 g; Glycyrrhizae Radix (*Glycyrrhiza uralensis *Fisch. ex DC.) 1 g; Atractylodis Rhizoma (*Atractylodes ovata *DC.) 3 g; Zingiberis Rhizoma (*Zingiber officinale *Roscoe) 1.5 g; Poria (*Poria cocos *Wolf) 3 g; Saussureae Radix (*Saussurea lappa *Clarke) 1 g; and Longanae Arillus (*Euphoria longana *Lam.) 3 g. All crude drugs used were purchased from Tochimoto Tenkaido (Osaka, Japan). The mixture of crude drugs for Kihi-to that represents one human daily dose was extracted with 600 ml of water at 100°C for 40 min and subsequently evaporated under reduced pressure and freeze-dried to 8.3 g of extract powder. This Kihi-to powder was then dissolved in water. Voucher specimen (Lot No.20060921) has been deposited at our laboratory.

The three-dimensional HPLC pattern of the constituents of Kihi-to is shown [see Additional file 1]. Kihi-to extract (1.0 g) was dissolved with methanol (20 mL) under ultrasonication for 30 min followed by centrifugation at 3,000 rpm for 5 min. The supernatant was filtrated with a membrane filter (0.45 μm) and then submitted for HPLC analysis (30 μL). The HPLC apparatus consisted of a Shimadzu LC 10A (analysis system software: CLASS-M10A ver. 1.64, Tokyo, Japan) equipped with a multiple wavelength detector (UV 200–400 nm) (Shimadzu SPD-M10AVP, diode array detector) and an auto injector (Shimadzu CTO-10AC). HPLC conditions were as follows: column, ODS (TSK-GEL 80TS, 250 × 4.6 mm i.d., TOSOH, Tokyo, Japan); eluant, (A) 0.05 M AcONH_4 _(pH 3.6) (B) 100% CH_3_CN (a linear gradient of 90% A and 10% B, which changed over 60 min to 0% A and 100%, B was used, followed by 100% B for a further 20 min); temperature, 40°C; flow rate, 1.0 mL/min.

### Water maze test

Male ddY mice (7 weeks old, Japan SLC) were housed with free access to food and water, and were kept in a controlled environment (22 ± 2°C, 50 ± 5% humidity, 12-h light cycle starting at 7:00 am). Animals were handled in accordance with the Guidelines for the Care and Use of Laboratory Animals of the University of Toyama, and all protocols were approved by the Animal Care Committee of the University of Toyama. Aβ(25–35) was dissolved in saline at a concentration of 5 mM and incubated at 37°C for 4 days to allow for fibril formation. The mice were anesthetized, and Aβ(25–35) (25 nmol in 5 μl) or the reverse non-active sequence, Aβ(35–25) (25 nmol in 5 μl), was injected into the right ventricle using the following stereotaxic coordinates from the bregma (mm): A -0.22, L -1.0, and V 2.5. Ten days after an i.c.v. injection of Aβ(25–35), Kihi-to (100 mg/kg/day, p.o.), or the vehicle (tap water, p.o.) was administered once daily for 3 days. We previously confirmed that mice injected by Aβ(35–25) showed similar memory activities to saline-injected mice. At present study, we used Aβ(35–25) for making a control group to indicate that Aβ(25–35)-induced memory deficits were sequence-dependent phenomena.

The Morris water maze test was performed as follows: purple-colored water was poured into a round tank (diameter, 122 cm; height, 28 cm), and a purple platform (diameter, 12 cm) was placed 1.2 cm below the water level in the middle of a fixed quadrant. The water temperature was adjusted to 21–23°C. Memory acquisition test was performed four times daily (60 min intervals between tests) for 5 days. The mice were allowed to swim freely (time limit; 60 s) to seek an invisible platform and were left for an additional 30 s on the platform. Time spent to reach to the platform was defined as the escape latency. The platform position was not moved during all trials. The pattern for the rotation of the start position was changed daily. Mice failing to find the platform after 60 s were manually placed on the platform. Memory-retention tests were performed 3 days after the last training session, that is, 8 days after the discontinuation of drug administration. The platform was removed, and each mouse was allowed a free 60-s swim. The number of crossings over the point where the platform had been located was counted. Swimming performance was recorded by a digital camera and analyzed by a tracking system, EthoVision 3.0 (Noldus Information Technology, Wageningen, The Netherlands).

### Novel object recognition test

Mice underwent the novel object recognition test at four days after the water maze retention test (i.e., 12 days after discontinuation of drug administration). Object A (a black vase) and object B (a glass box) were placed at a fixed distance within in a round field (diameter, 58 cm; height, 26.5 cm). A mouse was then placed at the opposite edge of the field, and the number of times it made contact with the two objects was recorded during a 5-min period (training session). Mice were then placed back into the same field 10 min after the training session, in which one of the familiar objects used during the training session was replaced with a novel object C (a white ball). The mice were then allowed to explore freely for 5 min and the number of times they made contact with each object was recorded (test session). A preference index, defined as the ratio of the number of times a mouse made contact with any of the objects (training session) or the novel object (test session) over the total number of times the mouse made contact with both objects, was used to measure cognitive function.

### Immunohistochemistry

Four days after the novel object recognition test, mice were killed by decapitation. The brains were quickly removed from the skull and frozen in powdered dry ice. The brains were cut in 12-μm coronal sections using a cryostat (CM3050S, Leica, Heidelberg, Germany), and the slices were fixed with 4% paraformaldehyde and stained with a monoclonal antibody against phosphorylated NF-H, MAP2, synaptophysin or MBP. Alexa Fluor 488-conjugated goat anti-mouse IgG and Alexa Fluor 568-conjugated goat anti-rabbit IgG were used as secondary antibodies. The staining and quantification were carried out under exactly similar conditions. The fluorescent images were captured using a fluorescent microscope (AX-80, Olympus, Tokyo, Japan) at 661 μm × 878 μm (striatum area) or 320 μm × 425 μm (other areas), and 3 – 5 sets of serial brain slices from 3 mice were used to capture the images for each treatment. The measuring points were selected with 10–25 squares (fixed size of a square: 10 × 10 μm) to cover the whole area of each region or subregion (e.g., the molecular layer of the dentate gyrus). The background intensity was determined by staining slices without each first antibody. Fluorescent intensities of immuno-positive areas (after subtracting the background intensity) in those squares were quantified using ATTO densitography (ATTO, Tokyo, Japan).

### Primary culture

Embryos were removed from pregnant Sprague-Dawley rats (Japan SLC, Shizuoka, Japan) at 17–18 days of gestation. The cortices were dissected, and the dura mater was removed. The tissues were minced and dissociated and then grown in cultures with Neurobasal medium including 12% horse serum, 0.6% D-glucose and 2 mM L-glutamine on 8-well chamber slides (Falcon, Franklin Lakes, NJ, USA) coated with 5 μg/ml poly-D-lysine at 37°C in a humidified incubator with 10% CO_2_. When Aβ(25–35) or other compounds were added, half of the medium in each well was replaced with fresh medium containing 2% B-27 supplement without serum. The time schedules of the experiments are illustrated at the bottom of each respective figure.

### Immunocytochemistry for measures of neurite length and expressions of calpain and calpastatin

Rat cortical neurons were cultured in 8-well chamber slides at a density of 1.45 – 2.2 × 10^5 ^cells/cm^2^. For measuring lengths of axons and dendrites, the cells were treated with 10 μM Aβ(25–35) for 4 days, followed by the addition of the extract, mouse β-NGF, or vehicle (0.1% DMSO). Five days later, the cells were fixed with 4% paraformaldehyde and then immunostained with a monoclonal antibody against phosphorylated NF-H (1:1000) as an axonal marker or a monoclonal antibody against MAP2 (1:1000) as a dendritic marker. Alexa Fluor 488-conjugated goat anti-mouse IgG (1:200) was used as a second antibody. For measuring levels of calpain and calpastatin, the cells were incubated with 10 μM Aβ(25–35) and 0.1 μg/ml Kihi-to or 1 nM MDL28170 simultaneously for 2, 8, 24 96 h. The cells were fixed with 4% paraformaldehyde and then double-immunostained with a monoclonal antibody against μ-calpain (1:500) and polyclonal antibody against MAP2, or a polyclonal antibody against calpastatin (1:500) and a monoclonal antibody against MAP2. Alexa Fluor 488-conjugated goat anti-mouse IgG (1:200) and Alexa Fluor 568-conjugated goat anti-rabbit IgG (1:200) was used as second antibodies. The fluorescent images were captured by a fluorescent microscope (AX-80) at 320 μm × 425 μm, and four images were captured per treatment. The lengths of neurites that were positive for phosphorylated NF-H or MAP2 were measured using an image analyzer Neurocyte (Kurabo, Osaka, Japan) which detects neurite lengths. The total length of axons or dendrites was divided by cell numbers in the identical area to show an average length per cell. Expression levels of calpain and calpastatin in MAP2-positive neuronal cell bodies were quantified using ATTO densitography as described in a Immunohistochemistry method.

### Cell viability assessment

Rat cortical neurons were cultured in 8-well chamber slides at a density of 1.45 × 10^5 ^cells/cm^2^. Cell viability was determined by calcein staining. Cells on 8-well chamber slides were rinsed by phosphate-buffer saline (PBS), and were incubated with 6 μM calcein AM (Dojindo, Kumamoto, Japan) for 40 min at 37°C. After rinsing by PBS, cells were fixed by 4% paraformaldehyde and mounted. Fluorescence images and bright field images were simultaneously captured (four images per treatment) by AX-80 microscope. The percentage of dead cell was valued as the ratio of dead cells (calcein-negative) to total cells. The total cell number was counted in bright-field photos.

### Ca^2+ ^imaging

After neuronal cells were incubated with fluo-4 AM (8 μM; Dojindo) in serum-free medium for 40 min at 37°C, the cells were washed and incubated further without fluo-4 AM in serum-free medium for 30 min at room temperature. Then, medium was replaced with HEPES buffer (of composition, in mM, NaCl 145; MgSO_4 _1; KCl 2.5; D-glucose 10; CaCl_2_1; HEPES 10; pH 7.3). Cells were placed on a heated (37°C) stage and viewed using a confocal laser scanning microscope (TCS-SP5, Leica Microsystems, Tokyo, Japan). Excitation and emission wavelengths ware 488 nm and 520 nm, respectively. Time-lapse images were recorded every 5 s from 10 s before and up to 30 s after the drug administration. Peak fluorescence change was calculated as relative change from baseline using the formula Δ*F*/*F*% = (*F *- *F*_0_)/*F*_0 _* 100.

### Statistical Analysis

Statistical comparisons were performed using one-way analysis of variance (ANOVA), repeated measures two-way ANOVA followed by Holm-Sidak *post hoc *test, or paired *t*-test. Values of *p *< 0.05 were considered significant. The means of the data are presented together with the SE.

## Results

### Kihi-to ameliorates Aβ(25–35)-induced impairments in spatial memory and object recognition

**Figure 1:**
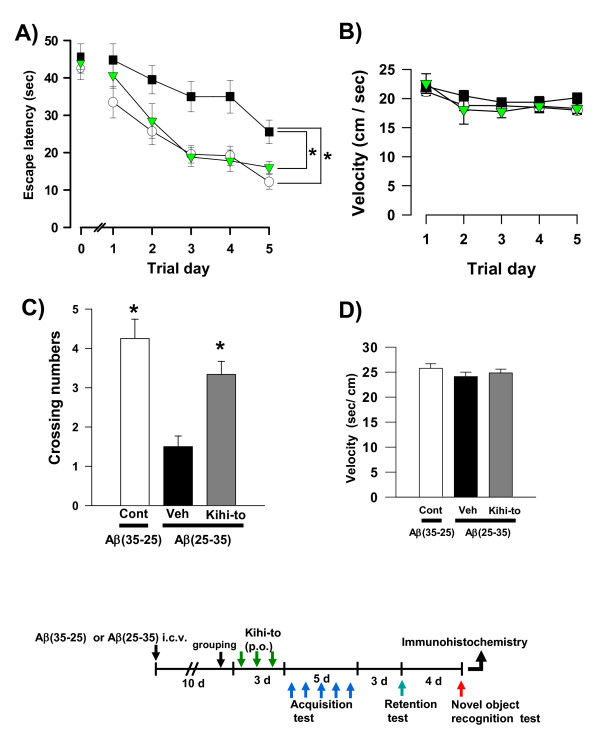
**Effects of Kihi-to on Aβ(25–35)-induced spatial memory deficits**. Aβ(25–35) (25 nmol) was injected into the right lateral ventricle of mice. From ten days after the injection, mice were administered vehicle (Veh, water by p.o.; DMSO by i.v., *n *= 10; squares) or Kihi-to (100 mg/kg B.W., p.o., *n *= 9; triangles) for 3 days. The control mice (Cont, *n *= 8; circles) were injected with a reverse peptide, Aβ(35–25), and then administered vehicle. After that, memory acquisition tests were continued for 5 days in a Morris water maze (A). Escape latencies to a hidden platform were measured. Three days after the last trial of the memory acquisition test, the memory retention test was performed (C). The number of crossings over the position at which the platform had been located was measured for 60 s. Swimming velocities of mice in the memory acquisition test (B) and the retention test (D) are shown. *p < 0.05 vs. Veh. (a: Repeated measures two-way ANOVA followed by Holm-Sidak *post hoc *test, c: one-way ANOVA followed by Holm-Sidak *post hoc *test).

The densities of neurites and synapses are decreased in the hippocampus and cerebral cortex of mice 7 days after i.c.v. administration of Aβ(25–35), and these deficits persist for at least 30 days (unpublished data). A pretest in a water maze was performed 9 days after i.c.v. administration of Aβ(25–35). At that time, the escape latency in Aβ(25–35)-injected mice was already slightly longer than that in Aβ(35–25)-injected mice (Figure 1A, Trial day 0). Mice were then administered Kihi-to orally (p.o.) every day for 3 days, beginning 10 days after i.c.v. administration of Aβ(25–35), and Morris water maze testing was performed. After completing the memory acquisition test each day for 5 days, the mice rested for 3 days. Then, mice were subjected to a memory retention test, wherein the number of crossings over the platform position was counted.

The time to reach the invisible platform decreased with each trial day in all groups. Aβ(25–35)-injected mice receiving vehicle showed a slow decrease in the escape latency when compared with Aβ(35–25)-injected control mice. By contrast, Kihi-to-treated mice that were injected Aβ(25–35) showed a relatively rapid decrease in escape latency (Figure 1A). Repeated measures two-way ANOVA revealed significant group effects (Kihi-to *vs*. Aβ(25–35)-injected and vehicle-treated, F(1, 68) = 10.89, *P *= 0.004). Swimming velocities in the memory acquisition test were not different when comparing these three groups (Figure 1B).

The number of crossings in the retention test was significantly lower in Aβ(25–35)-injected mice receiving vehicle than in control mice (Figure 1C). The number of crossings was significantly higher in the Aβ(25–35)-injected mice receiving Kihi-to than those receiving vehicle. Swimming velocities in the memory retention test were not different when comparing these three groups (Figure 1D).

**Figure 2:**
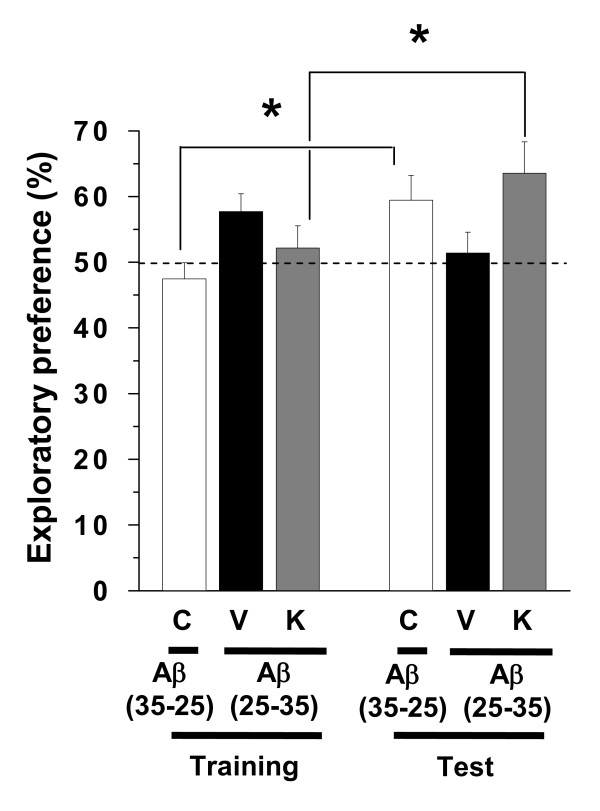
**Effects of Kihi-to on Aβ(25–35)-induced object recognition memory deficits**. Aβ(25–35) (25 nmol) was injected into the right lateral ventricle of mice. From ten days after the injection, mice were administered vehicle (**V**, water by p.o.; DMSO by i.v., *n *= 10) or Kihi-to (**K**, 100 mg/kg B.W., p.o., *n *= 9) for 3 days. The control mice (**C**, *n *= 8) were injected with a reverse peptide, Aβ(35–25) and then administered vehicle. Twelve days after the last drug administration, a novel object recognition test was performed (see drug administration schedule in Figure 1). A mouse was placed in the field, and the number of times it made contact with the two objects was recorded for 5 min (training session). Mice were placed back into the same field 10 min after the training session, in which one of the familiar objects used during the training session was replaced with a novel object. The mice were then allowed to explore the area freely for 5 min, and the amount of time spent exploring each object was recorded (test session). The preference index was defined as the ratio of the number of times a mouse made contact with any one of the objects (training session) or the novel object (test session) over the total number of times the mouse made contact with both objects. *p < 0.05 vs. Veh. (paired *t*-test).

Visual recognition memory was assessed using a novel object recognition test. Compared with the training session, control mice and Aβ(25–35)-injected mice receiving Kihi-to showed significantly more frequent exploratory behaviors to a novel object than a familiar object (Figure 2). Used dose of Kihi-to, 100 mg/kg/day is similar to human daily dose (approximately 125 mg/kg/day), and was shown as a maximal effective dose by our previous experiment.

### Kihi-to increases the density of neuritis, synapses and myelin in the brain of Aβ(25–35)-injected mice

**Figure 3:**
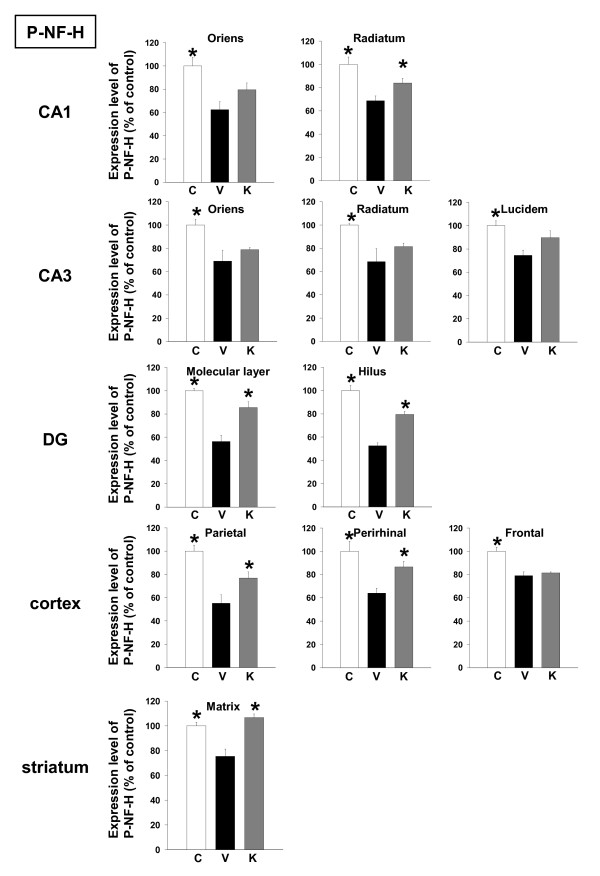
Effects of Kihi-to on Aβ(25–35)-induced decreases in axons. Aβ(25–35) (25 nmol) was injected into the right lateral ventricle of mice. From ten days after the injection, mice were administered vehicle (**V**, water by p.o.; DMSO by i.v.) or Kihi-to (**K**, 100 mg/kg B.W., p.o.) for 3 days. The control mice (**C**) were injected with a reverse peptide, Aβ(35–25) and then administered vehicle. After the novel object recognition test (Figure 2), brain slices were immunostained with phosphorylated neurofilament-H (P-NF-H) antibody. P-NF-H-positive areas were quantified in the stratum oriens and stratum radiatum in CA1, the stratum oriens, stratum radiatum and stratum lucidum in CA3, the molecular layer and hilus in the dentate gyrus (DG), the parietal cortex, perirhinal cortex, frontal cortex, and the striatum. *p < 0.05 vs. Veh. *n *= 3. (one-way ANOVA followed by Holm-Sidak *post hoc *test).

Following the memory retention test, the levels of P-NF-H, MAP2, synaptophysin and myelin basic protein (MBP, a myelin marker) were measured in the brains of the mice by immunohistochemistry. The brain regions assessed included three cortical regions (frontal cortex, parietal cortex and perirhinal cortex), seven subregions in three hippocampal regions (CA1, CA3, and dentate gyrus), and the striatum. In case of MBP, cortical regions (e.g., striatum, corpus callosum) were selected for assessment, as those regions are myelin-rich. Expression levels of P-NF-H were decreased in all regions of Aβ(25–35)-injected mice receiving vehicle compared to control mice, and administration of Kihi-to resulted in significant increases in P-NF-H expression levels in CA1 radiatum, dentate gyrus, parietal cortex, perirhinal cortex and the striatum (Figures 3 and 7).

**Figure 4:**
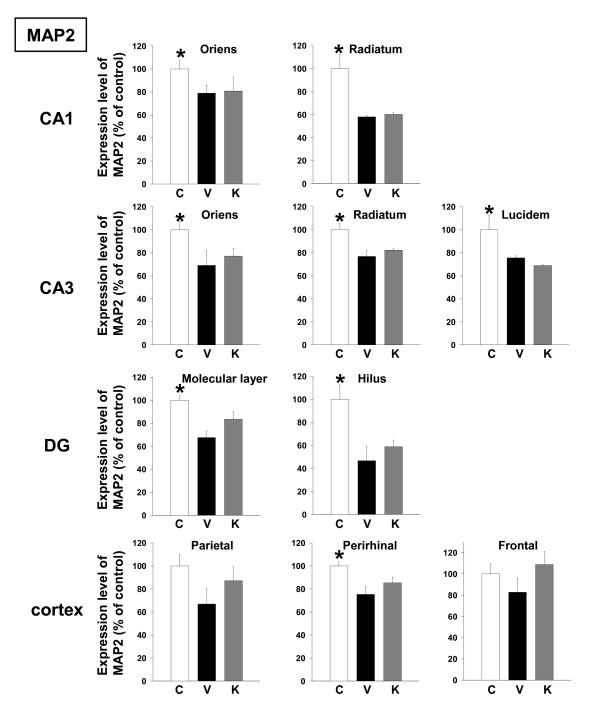
Effects of Kihi-to on Aβ(25–35)-induced decreases in dendrites. Aβ(25–35) (25 nmol) was injected into the right lateral ventricle of mice. From ten days after the injection, mice were administered vehicle (**V**, water by p.o.; DMSO by i.v.) or Kihi-to (**K**, 100 mg/kg B.W., p.o.) for 3 days. The control mice (**C**) were injected with a reverse peptide, Aβ(35–25) and then administered vehicle. After the novel object recognition test (Figure 2), brain slices were immunostained with MAP2 antibody. MAP2-positive areas were quantified in the stratum oriens and stratum radiatum in CA1, the stratum oriens, stratum radiatum and stratum lucidum in CA3, the molecular layer and hilus in the dentate gyrus (DG), and the parietal, perirhinal and frontal cortex. *p < 0.05 vs. Veh. *n *= 3. (one-way ANOVA followed by Holm-Sidak *post hoc *test).

Expression levels of MAP2 were also decreased in all brain regions of Aβ(25–35)-injected mice receiving vehicle compared to control mice, and administration of Kihi-to tended to increase MAP2 expression level in the dentate gyrus and cortex (Figures 4 and 7).

**Figure 5:**
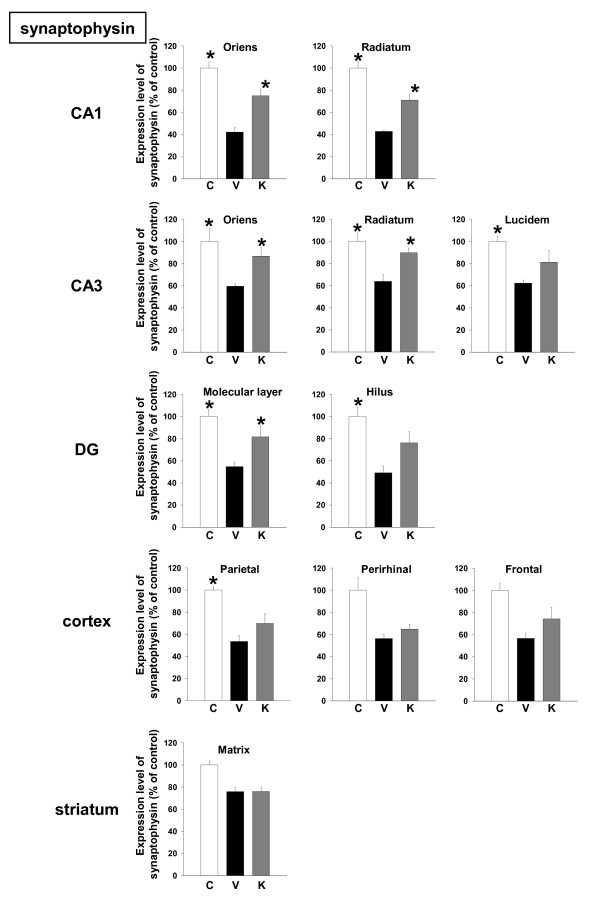
Effects of Kihi-to on Aβ(25–35)-induced decreases of synapses. Aβ(25–35) (25 nmol) was injected into the right lateral ventricle of mice. From ten days after the injection, mice were administered vehicle (**V**, water by p.o.; DMSO by i.v.) or Kihi-to (**K**, 100 mg/kg B.W., p.o.) for 3 days. The control mice (**C**) were injected with a reverse peptide, Aβ(35–25) and then administered vehicle. After the novel object recognition test (Figure 2), brain slices were immunostained with synaptophysin antibody. Synaptophysin-positive areas were quantified in the stratum oriens and stratum radiatum in CA1, the stratum oriens, stratum radiatum and stratum lucidum in CA3, the molecular layer and hilus in the dentate gyrus (DG), the parietal cortex, perirhinal cortex, frontal cortex, and the striatum. *p < 0.05 vs. Veh. *n *= 3. (one-way ANOVA followed by Holm-Sidak *post hoc *test).

Expression levels of synaptophysin were decreased in all brain regions of Aβ(25–35)-injected mice receiving vehicle, and administration of Kihi-to resulted in significant increases in synaptophysin expression levels in CA1, the oriens and radiatum in CA3, the molecular layer in dentate gyrus (Figures 5 and 7).

**Figure 6:**
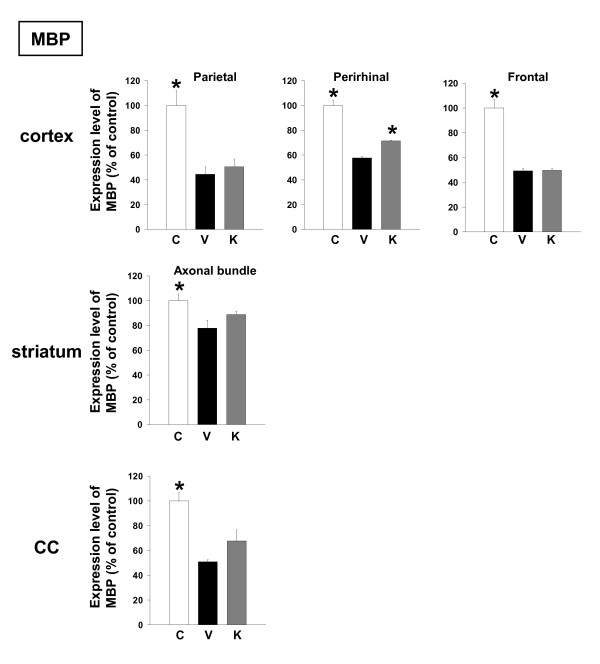
Effects of Kihi-to on Aβ(25–35)-induced decreases of myelin. Aβ(25–35) (25 nmol) was injected into the right lateral ventricle of mice. From ten days after the injection, mice were administered vehicle (**V**, water by p.o.; DMSO by i.v.) or Kihi-to (**K**, 100 mg/kg B.W., p.o.) for 3 days. The control mice (**C**) were injected with a reverse peptide, Aβ(35–25) and then administered vehicle. After the novel object recognition test (Figure 2), brain slices were immunostained with myelin basic protein (MBP) antibody. MBP-positive areas were quantified in the striatum, corpus callosum, and the parietal, perirhinal and frontal cortex. *p < 0.05 vs. Veh. *n *= 3. (one-way ANOVA followed by Holm-Sidak *post hoc *test).

**Figure 7:**
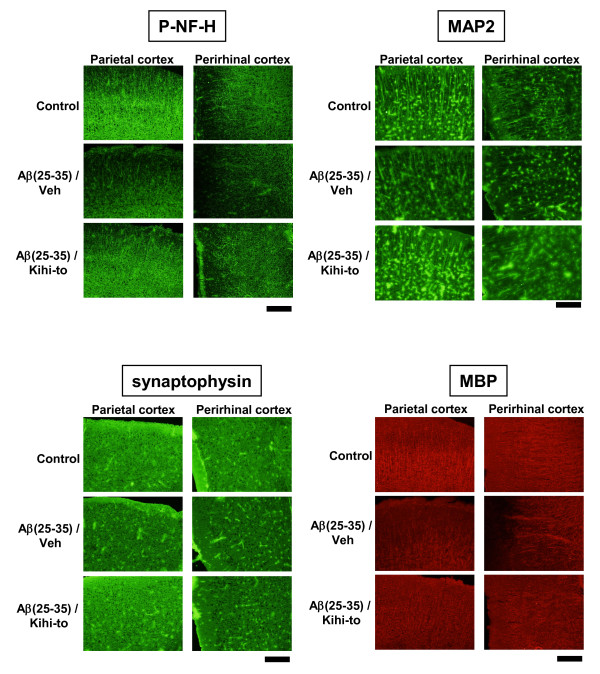
Effects of Kihi-to on Aβ(25–35)-induced decreases in axons, dendrites, synapses and myelins. Typical slice images of the parietal and perirhinal cortex were shown for P-NF-H, MAP2, synaptophysin and MBP. Scale = 100 μm.

Expression levels of MBP were decreased in the cortex, striatum axonal bundles and corpus callosum of Aβ(25–35)-injected mice receiving vehicle, and administration of Kihi-to resulted in an increase in MBP expression levels in the perirhinal cortex (Figures 6 and 7).

### Kihi-to reduces the calpain expression level in the brain of Aβ(25–35)-injected mice

**Figure 8:**
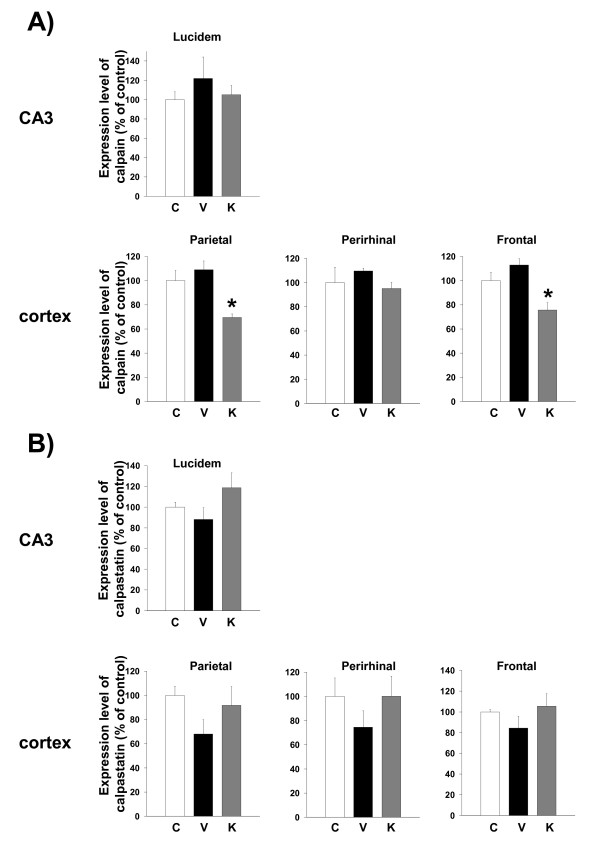
Effects of Kihi-to on Aβ(25–35)-induced increases of calpain and decreases in calpastatin. Aβ(25–35) (25 nmol) was injected into the right lateral ventricle of mice. From ten days after the injection, mice were administered vehicle (**V**, water by p.o.; DMSO by i.v.) or Kihi-to (**K**, 100 mg/kg B.W., p.o.) for 3 days. The control mice (**C**) were injected with a reverse peptide, Aβ(35–25) and then administered vehicle. After the novel object recognition test (Figure 2), brain slices were immunostained with μ-calpain (A) or calpastatin (B) antibody. Calpain-positive and calpastatin-positive areas were quantified in the stratum lucidum in CA3, and the parietal, perirhinal and frontal cortex. *p < 0.05 vs. Veh. *n *= 3. (one-way ANOVA followed by Holm-Sidak *post hoc *test).

Expression levels of calpain tended to be increased in the cortex and CA3 of Aβ(25–35)-injected mice receiving vehicle, and administration of Kihi-to resulted in a decrease in calpain expression levels in those regions especially in the parietal cortex and frontal cortex (Figure 8A). On the other hand, expression levels of calpastatin tended to be decreased in the cortex and CA3 of Aβ(25–35)-injected mice receiving vehicle, and administration of Kihi-to tended to increase the calpastatin expression levels in those regions (Figure 8B). There are not significant differences of calpain and calpastatin expressions between control and Aβ(25–35)-treated groups in other brain area.

### Kihi-to promotes axonal and dendritic extensions in damaged neurons

**Figure 9:**
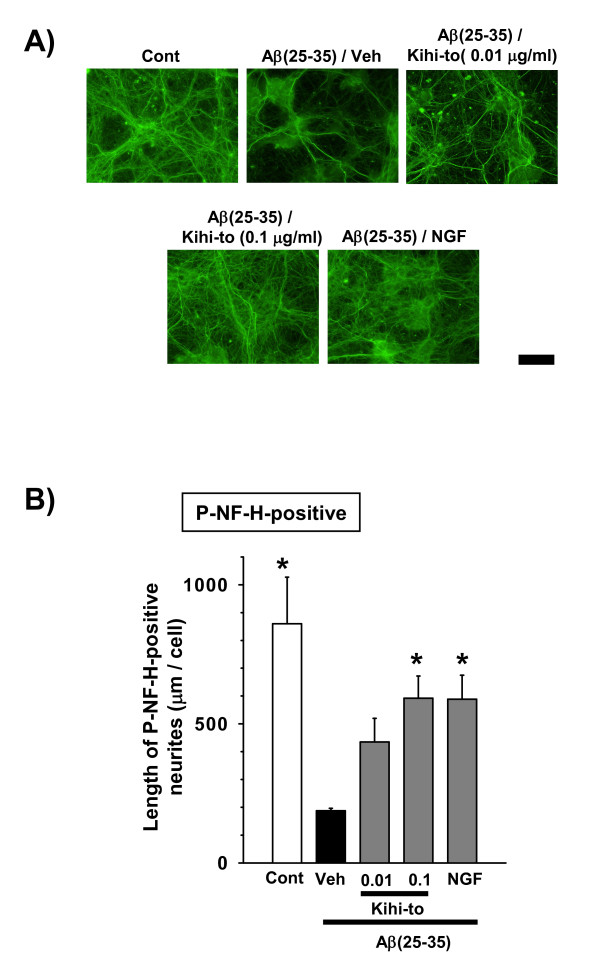
**Effects of Kihi-to on axonal extension following Aβ(25–35)-induced atrophy**. Cortical neurons were cultured for 4 days and then treated with or without (Cont) 10 μM Aβ(25–35). Four days after the administration of Aβ(25–35), the cells were treated with Kihi-to (0.01 and 0.1 μg/ml), 100 ng/ml of NGF, or vehicle (Veh). Five days after treatment, the cells were fixed and immunostained with an antibody against phosphorylated NF-H (A). The lengths of NF-H-positive neurites (B) were quantified for each treatment. *p < 0.05 vs. Veh, *n *= 4 (one-way ANOVA followed by Holm-Sidak *post hoc *test). Scale bar = 100 μm.

**Figure 10:**
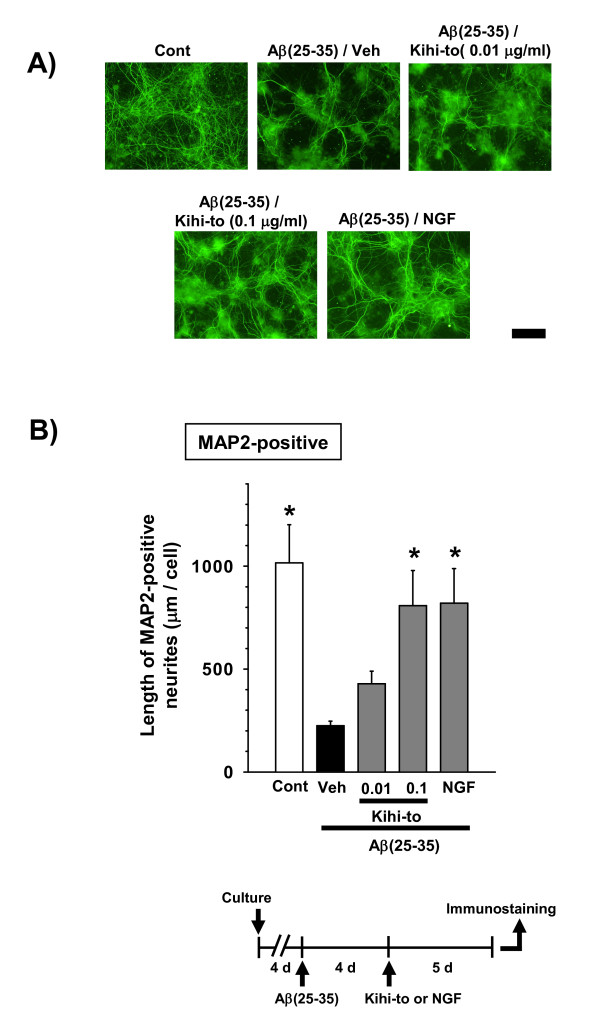
**Effects of Kihi-to on dendritic extension following Aβ(25–35)-induced atrophy**. Cortical neurons were cultured for 4 days and then treated with or without (Cont) 10 μM Aβ(25–35). Four days after the administration of Aβ(25–35), the cells were treated with Kihi-to (0.01 and 0.1 μg/ml), 100 ng/ml of NGF, or vehicle (Veh). Five days after treatment, the cells were fixed and immunostained with an antibody against MAP2 (A). The lengths of MAP2-positive neurites (B) were quantified for each treatment. *p < 0.05 vs. Veh, *n *= 4 (one-way ANOVA followed by Holm-Sidak *post hoc *test). Scale bar = 100 μm.

Kihi-to was administered 4 days after treatment with Aβ(25–35), and axon length (Figures 9A and 9B) or dendrite length (Figures 10A and 10B) was measured after an additional 5 days. Axon lengths and dendrite length were shorter in the cells treated with Aβ(25–35) followed by vehicle than in control cells. The axon and dendrite lengths were significantly longer in the Aβ(25–35)-treated cells when they were also treated with Kihi-to (0.1 μ g/ml) or NGF (100 ng/ml) than when they were treated with vehicle alone.

### Kihi-to attenuates Aβ(25–35)-induced cell damage

**Figure 11:**
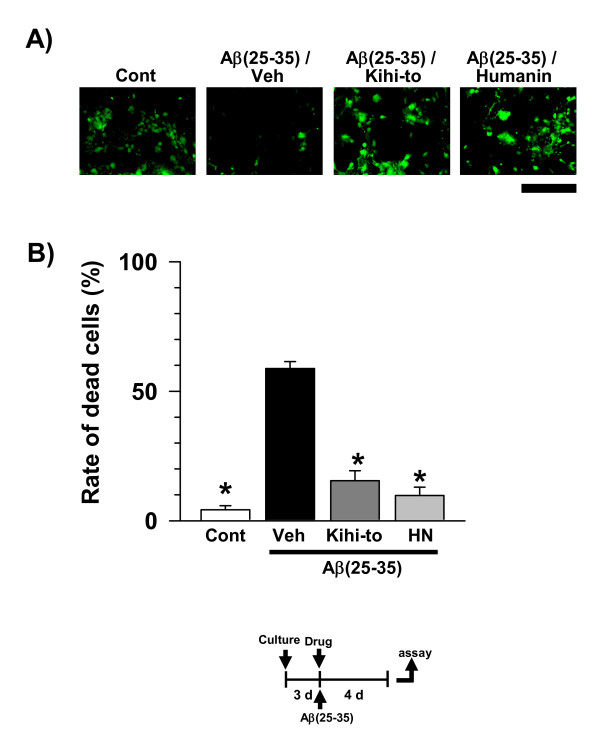
**Effects of Kihi-to on Aβ(25–35)-induced cell death in cortical neurons**. After cultivation for 3 days, the cortical neurons were treated with or without (Cont) Aβ(25–35). The cells were simultaneously treated with Kihi-to (1 μg/ml), [Gly^14^]-Humanin (10 nM) or vehicle (Veh). Four days after the treatment, cell viability was measured (B). Photographs show representative images (A). Scale = 100 μm. *p < 0.05 vs. Cont, *n *= 4 (one-way ANOVA followed by Holm-Sidak *post hoc *test).

Protective effects of Kihi-to on Aβ(25–35)-induced cell damage were investigated. Cortical neurons were treated by drug or vehicle (water) simultaneously with Aβ(25–35). Four days after that, cell viability was determined by measuring calcein uptake. [Gly^14^]-Humanin peptide was used as a positive control. This mutated form peptide has Gly^14 ^instead of Ser^14^, was shown to be effective on Aβ(25–35)-induced cell damage at lower dose (10 nM) compared with native form of the peptide [19]. Rate of damaged cells was increased by Aβ(25–35) treatment compared with control (Figure 11). At a dose of 0.1 μg/ml, Kihi-to suppressed the Aβ(25–35)-induced cell damage. Treatments with [Gly^14^]-Humanin (10 nM) inhibited the Aβ(25–35)-induced cell damage.

### Kihi-to inhibits the calpain expression

**Figure 12:**
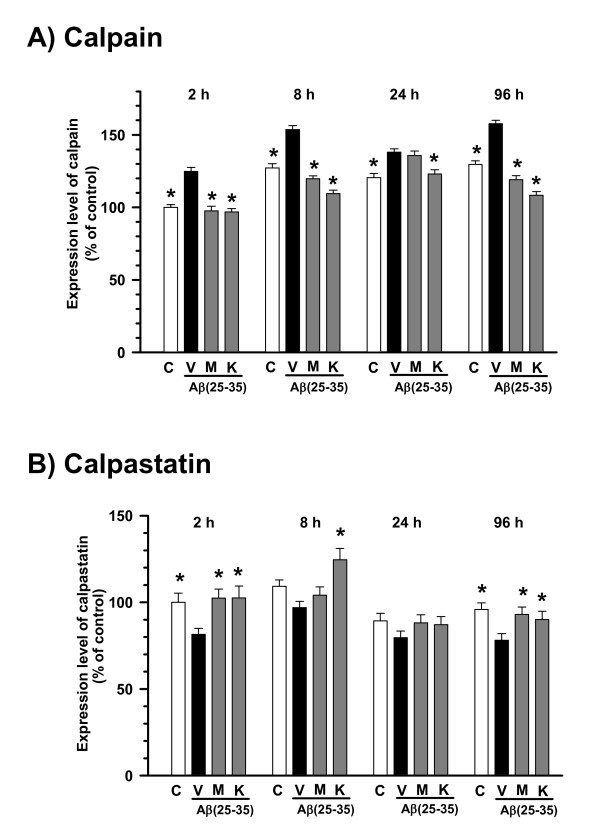
**Effects of Kihi-to on Aβ(25–35)-induced expression changes of calpain and calpastatin**. Cortical neurons were cultured for 2 days and then treated with or without (**C**) 10 μM Aβ(25–35). The cells were simultaneously treated with Kihi-to (0.1 μg/ml, **K**), MDL28170 (1 nM, **M**), or vehicle (**V**) for 2, 8, 24 and 96 h, and then double-immunostained for calpain and MAP2 or for calpastatin and MAP2. Expression levels of calpain (A) and calpastatin (B) in MAP2-positive cells (neurons) were quantified. *p < 0.05 vs. Veh, *n *= 40. (one-way ANOVA followed by Holm-Sidak *post hoc *test).

NF-H [20] and MAP2 [21] are cleaved by the Ca^2+^-dependent protease, μ-calpain. In addition, synaptophysin colocalizes with μ-calpain [22], and dynamin, a synaptic protein, is also a substrate of μ-calpain [23]. Further, the expression of μ-calpain is increased in the frontal cortex of Alzheimer's disease patients [24]. Therefore we investigated the expression levels of calpain and calpastatin in cortical neurons. We used intentionally mixed culture of neurons astrocytes oligodendrocytes and microglias to detect changes of neurons in a circumstance many glial cells surrounding neurons like in the brain. In the present experimental condition, a population of neurons was approximately 50%. Therefore, immunocytochemistry is more suitable than Western blotting for quantification of the neuron-specific expressions of calpain and calpastatin. Calpain-positive fluorescence in the cytosol of MAP2-positive neurons continuously increased at 2 h to 96 h after the treatment of Aβ(25–35) (Figure 10a). A cell-permeable calpain inhibitor, MDL28170 (1 nM) and Kihi-to (0.1 μg/ml) significantly inhibited this increase in calpain at any time points (Figure 12A). By contrast, the expression level of calpastatin, an endogenous inhibitor of calpain, in the cytosol of MAP2-positive neurons continuously decreased at 2 h to 96 h after the treatment of Aβ(25–35) (Figure 10b). MDL28170 (1 nM) and Kihi-to (0.1 μg/ml) increased the level of calpastatin at any time points (Figure 12B).

### Kihi-to inhibits and Aβ(25–35)-induced calcium elevation

**Figure 13:**
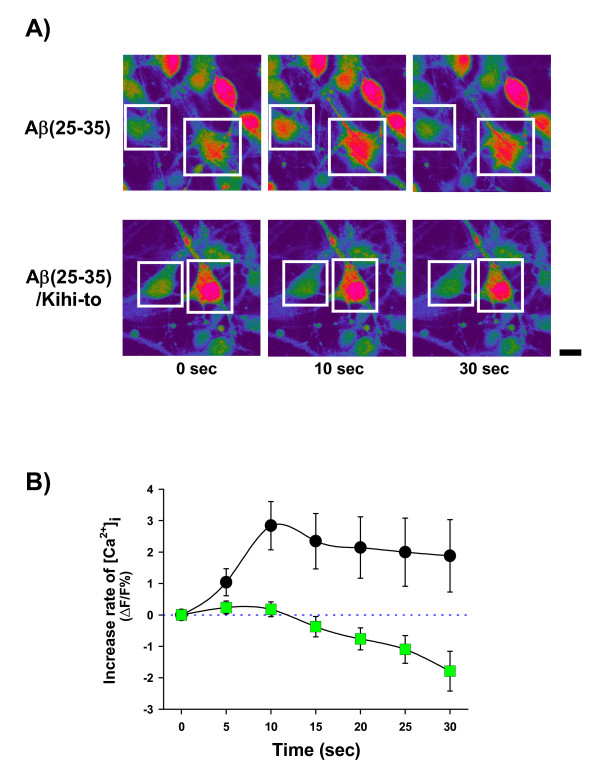
**Effects of Kihi-to on Aβ(25–35)-induced Ca^2+ ^influx**. Cortical neurons were cultured for 7 – 8 days and then loaded by fluo-4 AM (8 μM) for 40 min. After additional incubation for 30 min, cells were stimulated by 10 μM Aβ(25–35) alone (b; circles, n = 125) or 10 μM Aβ(25–35) and 1 μg/ml Kihi-to (b; squares, n = 83). Time-lapse images were captured every 5 s. Peak fluorescence change was calculated as relative change from baseline (Δ*F*/*F*%). Typical images were shown in (a). Scale bar = 10 μm.

Intracellular calcium ion ([Ca^2+^]i) was significantly increased in Aβ(25–35)-treated neurons (Figures 13A and 13B). In contrast, [Ca^2+^]i in Kihi-to (1 μg/ml) and Aβ(25–35)-treated neurons was completely inhibited. Repeated measures two-way ANOVA revealed a significant time and treatment interaction (Kihi-to *vs*. Aβ(25–35)-injected and vehicle-treated, F(6, 1236) = 2.657, *P *= 0.014).

## Discussion

In the present study, behavioral memory tests clearly showed that administration of Kihi-to for consecutive 3 days ameliorated the spatial and object recognition memories in Aβ(25–35)-injected mice (Figures 1 and 2). Kihi-to treatment slightly up-regulates the spatial memory also in normal mice (data not shown). Semi-quantification based on immunohistochemical comparisons suggested that Kihi-to treatment resulted in increases in the densities of axons especially in hippocampus CA1, the dentate gyrus, parietal cortex, perirhinal cortex, and striatum. Densities of presynapses were also increased in the hippocampus and the cortex. In the perirhinal cortex, the density of myelin was recovered. The hippocampus plays a key role for memory storage, and has connections to cortical and subcortical regions, the thalamus, hypothalamus and basal ganglia in the brain [25]. Alzheimer's disease model, Tg2576 mice show abnormalities in hippocampal morphology and physiology and displayed spatial memory but not object recognition [26]. Although abundant studies shows the hippocampus is crucial for memory acquisition and recalling, it is still in controversy whether the hippocampus is critical for familiarity recognition or not [27]. By contrast, the perirhinal cortex mediates spatial memory retention [28] and is also crucial for the discrimination and memorization of novel and familiar individual objects [29]. In addition, the parietal cortex is essential for long-term spatial memory [30] and object recognition [31] in rats. In combination with the present data, these observations suggest that Kihi-to can attenuate losses of axons, synapses and myelin in critical areas for memory recall and recognition.

*In vitro *experiments demonstrated that Kihi-to restored axonal and dendritic outgrowths (Figures 9 and 10) and inhibited cell death (Figure 11) in Aβ(25–35)-treated cultured cortical neurons. Although in immunohistochemistry of brain slices, increases in "densities" of P-NF-H-positive and MAP2-positive stainings were indicated in a Kihi-to-treated group, the increased densities may be resulted from at least in part elongations of axons and dendrites. Aβ(25–35) evoked neuritic abnormality and cell death in our experiments at cellular level. These two phenomena seem to be mediated by different cellular signaling pathways. Heredia et al. reported that Aβ(1–40) or Aβ(25–35) induced dramatic reduction in the axonal network and the dystrophy related to actin remodeling in the aberrant focal adhesion complex mediated by activations of LIM kinase and cofilin [32]. Aβ(1–42)-induced axonal degeneration was also inhibited by a calpain inhibitor in an apoptosis-independent manner [33]. By contrast, cell death triggered by Aβ seems to be mediated other cellular mechanisms. Aβ(1–40) and Aβ(25–35) evoke cultured cortical and hippocampal cell deaths associated with caspase-3 activation [34]. Caspase inhibitors blocks Aβ(1–42)-induced apoptosis [33]. Aβ(25–35)-induced cell death is also known to be mediated by c-Jun N-terminal kinase activation [35]. Therefore, Kihi-to may have inhibitory potentials against neuritic dystrophy and cell death possibly by multi mechanisms. As shown in Figure 13, Kihi-to antagonized Aβ(25–35) actions such as Ca^2+ ^entry. The Aβ-induced Ca^2+ ^efflux elicits activations of caspase-9 and caspase-3, resulting in neuronal apoptosis [36]. Simultaneously treated Kihi-to with Aβ(25–35) inhibited neuronal death (Figure 11) and Ca^2+ ^entry (Figure 13). These suggest that Kihi-to may repress neuronal death at least in part by Aβ-induced Ca^2+ ^entry. In our experiments [37], Aβ injection into the brain elicits no apparent neuronal death although treatment with Aβ induces cell death in culture. However, the neuroprotective effect of Kihi-to must be advantageous in the patient's brain where neuronal death is severely progressing.

NF-H and MAP2 are substrates of the Ca^2+^-dependent cysteine protease, calpain [20,21,30], the levels of calpain are increased in the brains of patients with Alzheimer's disease [22]. Calpain inhibition enhances neurite outgrowth in neuroblastoma SH-SY-5Y cells [38] and Aβ(1–42)-treated sympathetic neurons [33], and increases growth cone motility [39]. Therefore, we measured effects of Kihi-to on the levels of calpain and calpastatin, an intrinsic inhibitor of calpain. Interestingly, Kihi-to showed sustained inhibition of the calpain level and increase in the calpastatin level in neurons at least for 96 h (Figure 12). Aβ(25–25)-induced calpain increase and calpastatin decrease occurred also in GFAP-positive astrocytes, and those were inhibited by Kihi-to-treatment (data not shown). Further, the increase in calpain and decrease in calpastatin were detected even at 26 days after injection of Aβ(25–35) in mice brain, and those were attenuated by Kihi-to treatment (Figure 8). The sustained activation of the calpain system by Aβ(25–35) in very interesting. By contrast an increase in calpain in Alzheimer's disease brain [22], the calpastatin expression is markedly reduced in the neocortex in Alzheimer's disease [40]. The expression of calpastatin could be regulated by calpain activity since calpastatin is cleaved by calpain [41]. Further, previous studies have demonstrated an inverse correlation between calpain and calpastatin expression levels in the brain of Tg2576, a mouse model of Alzheimer's disease [42]. Increased calpastatin inhibit Aβ(1–42)-induced axonal degeneration in rat sympathetic neurons [33], suggesting that inhibition of the calpain system may lead to extension of neurites. The present results showed that Kihi-to may inhibit the calpain system both in case of simultaneous treatment with Aβ(25–35) *in vitro *(Figure 12) and post-treatment *in vivo *(Figure 8). Although it is not known how Kihi-to regulates the expressions of calpain and calpastatin, inhibitory potential of Kihi-to against the calpain system may effect positively on neuritic remodeling.

## Conclusion

In conclusion Kihi-to clearly improved the memory impairment and losses of neurites and synapses. Dysregulation of expression levels of calpain and calpastatin by Aβ(25–35) were also attenuated by Kihi-to. Natural medicines involving Japanese-Chinese traditional herbal drugs, are not necessary in a position of just complementary medicines with only moderate effects. They are sometimes show clear-cut ameliorative effects. In our preliminary data, all twelve crude drugs which composed Kihi-to are needed to reveal the effect of neurite extension, suggesting that we would lose sight of clear effects of Kihi-to during isolation of active compounds. Therefore, we are now inclusively investigating target molecules of Kihi-to without isolation active constituents in it. After determination of several key target molecules, corresponding compounds for each molecule could be analyzed. Kihi-to is already available medicine to be prescribed by medical doctors in Japan. Although further basic researches and a lot of clinical studies should be needed, Kihi-to is a quite attracting candidate for anti-dementia drug.

## Competing interests

The authors declare that they have no competing interests.

## Authors' contributions

CT contributed in acquisition, analysis and interpretation of the data and wrote the manuscript. RN contributed in acquisition, analysis and interpretation of the data. EJ contributed in acquisition, analysis and interpretation of the data. All authors have read and approved the final version of the manuscript.

## Pre-publication history

The pre-publication history for this paper can be accessed here:



## Supplementary Material

Additional file 1 HPLC profile of Kihi-to and UV spectra of its constituents. The data provided 3D HPLC profiles of constituents of Kihi-to. Click here for file
