# Supplementary figures and images for: Kihi-to, a herbal traditional medicine, improves Abeta(25–35)-induced memory impairment and losses of neurites and synapses
Source: BMC Complement Altern Med. 2008 Aug 16;8:49. doi: 10.1186/1472-6882-8-49 (PMC2532680; doi:10.1186/1472-6882-8-49)

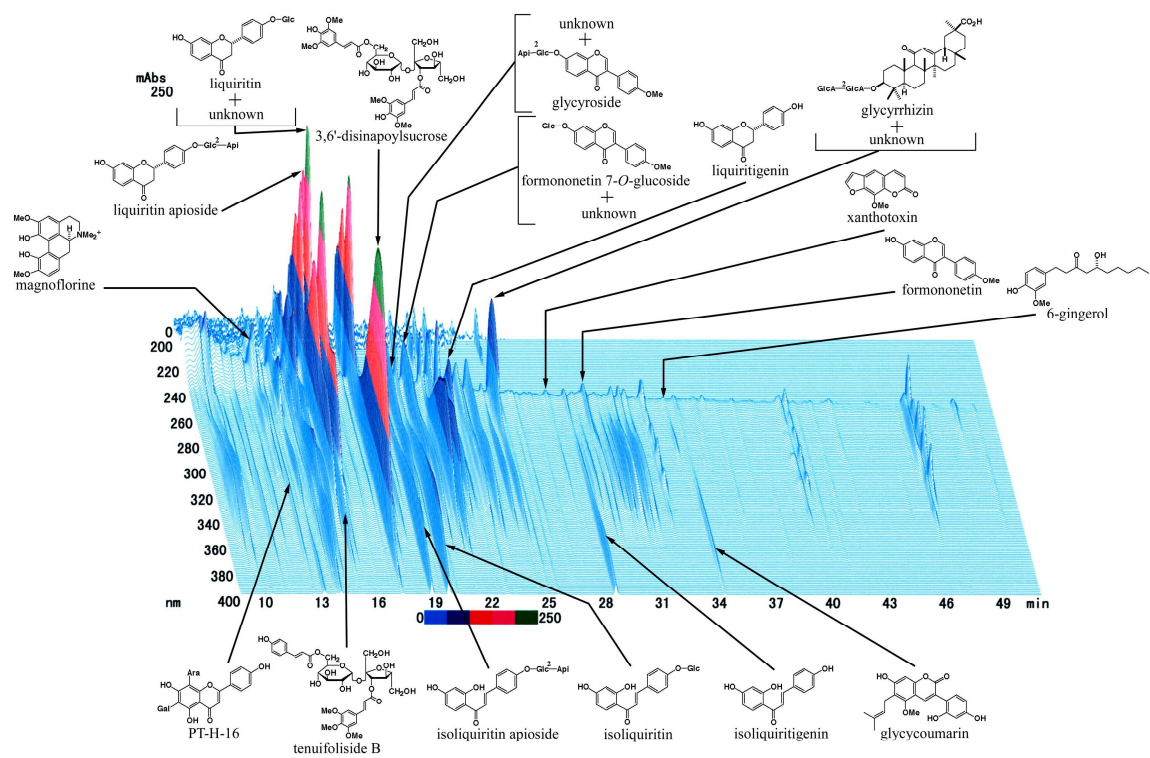

Supplement: Additional file 1 — HPLC profile of Kihi-to and UV spectra of its constituents. The data provided 3D HPLC profiles of constituents of Kihi-to. [file 1472-6882-8-49-S1.pdf]
